# 阿伐替尼治疗系统性肥大细胞增多症合并骨髓增生异常综合征/骨髓增殖性肿瘤1例报告并文献复习

**DOI:** 10.3760/cma.j.cn121090-20241011-00389

**Published:** 2025-05

**Authors:** 语薇 唐, 丽娟 潘, 富慧 李, 志坚 肖, 泽锋 徐

**Affiliations:** 1 中国医学科学院血液病医院（中国医学科学院血液学研究所），血液与健康全国重点实验室，国家血液系统疾病临床医学研究中心，细胞生态海河实验室，天津 300020 State Key Laboratory of Experimental Hematology, National Clinical Research Center for Blood Diseases, Haihe Laboratory of Cell Ecosystem, Institute of Hematology & Blood Diseases Hospital, Chinese Academy of Medical Sciences & Peking Union Medical College, Tianjin 300020, China; 2 天津医学健康研究院，天津 301600 Tianjin Institutes of Health Science, Tianjin 301600, China

## Abstract

系统性肥大细胞增多症（SM）合并骨髓增生异常综合征/骨髓增殖性肿瘤（MDS/MPN）是一种较为罕见的髓系肿瘤。阿伐替尼是一种高效的KIT D816V选择性抑制剂，被批准用于治疗进展型系统性肥大细胞增多症（AdvSM）。我们报道1例应用阿伐替尼治疗SM合并MDS/MPN患者，SM得到持续完全缓解且KIT D816V突变持续转阴，最终因进展为慢性粒-单核细胞白血病而死亡。虽然新的靶向药物阿伐替尼显著提高了SM的疗效，但对于SM合并相关血液肿瘤（SM-AHN）患者，其伴随的血液肿瘤的疗效可能是影响其长期生存和无疾病进展生存期的主要因素。本文通过相关文献复习，以期指导对该类疾病的临床诊治。

## Abstract (en)

Systemic mastocytosis（SM）with an associated myelodysplastic/myeloproliferative neoplasm (MDS/MPN) is a rare subtype of myeloid neoplasms. Avapritinib, a potent and selective inhibitor of KIT D816V, is approved for treating advanced systemic mastocytosis（AdvSM）. We report a case of a patient with SM and an associated MDS/MPN treated with avapritinib. The patient achieved sustained complete remission（CR）of SM, with persistent molecular negativity for the KIT D816V mutation, but ultimately succumbed to disease progression to chronic myelomonocytic leukemia（CMML）. Although avapritinib, a novel targeted therapy, has significantly improved outcomes for SM, the efficacy of treatment for the associated hematologic neoplasm in patients with SM-AHN may be the primary determinant of long-term overall survival and progression-free survival. This report includes a review of relevant literature to provide insights into the clinical diagnosis and management of this rare entity.

系统性肥大细胞增多症（SM）是一种罕见的异质性肿瘤，以过度增殖的肥大细胞在真皮组织以外的一个或多个组织、器官或系统浸润为特征，具有侵袭性，预后较差[1]–[2]。SM患者通常表现为皮肤瘙痒、反复性过敏反应、乏力、眩晕等肥大细胞活化症状及血细胞减少、肝脾肿大等症状。SM与KIT基因的体细胞突变密切相关，超过90％的患者可检出KIT突变，以D816V突变最为常见[3]。近年来，阿伐替尼（Avapritinib）作为KIT D816V的选择性抑制剂，极大地提高了SM临床疗效。我们用阿伐替尼治疗1例SM合并骨髓增生异常综合征/骨髓增殖性肿瘤（MDS/MPN）患者，SM得到持续缓解，最终因MDS/MPN疾病进展而死亡，现报道如下并对相关文献进行复习。

## 病例资料

患者，男，61岁，主因“发现脾大3年余，头晕乏力，腹胀加重6个月”就诊于我院。入院时查血常规示：RBC 2.05×10^12^/L，HGB 70 g/L，WBC 29.19×10^9^/L，PLT 141×10^9^/L。入院查体：生命体征平稳，中度贫血貌，脾脏可触及肿大，左肋缘下6 cm，余无明显异常。外周血涂片分类计数：原始细胞0.010，中性中幼粒细胞0.060，中性晚幼粒细胞0.010，中性杆状核粒细胞0.010，中性分叶核粒细胞0.340，嗜酸性粒细胞0.150，嗜碱性粒细胞0.330，成熟淋巴细胞0.050，成熟单核细胞0.040。髂骨穿刺骨髓涂片分类：增生明显活跃，粒系占0.735，红系占0.145，原始细胞占0.015，粒系形态大致正常，红系以中晚幼红细胞为主；全片共见巨核细胞21个。骨髓组织病理学检查：骨髓增生极度活跃（约90％），粒红比例增大，粒系各阶段细胞可见，嗜酸性粒细胞易见，红系以中晚幼红细胞为主，巨核细胞不少，分叶核为主，可见多灶纤维组织增生，网状纤维染色MF-2级。免疫组化：CD117（+），CD25（+），CD30（少量+），CD2（–），CD3（少量+），CD34（–），CD123（部分+）。其内可见肥大细胞，呈多灶性致密浸润，表达CD117，异常表达CD25，其中梭形的肥大细胞占比大于25％，结论：符合SM（图1）。细胞组织化学染色：中性粒细胞碱性磷酸酶（N-ALP）阳性率10％，阳性指数13；铁染色：环形铁粒幼红细胞阳性率45％。CD41免疫组织化学染色：全片巨核680个，其中正常巨核细胞427个，双核巨核细胞29个，多核巨核细胞13个，大单圆核小巨核细胞121个，单圆核小巨核细胞71个，双圆核小巨核细胞8个，多圆核小巨核细胞1个，淋巴样小巨核细胞10个。染色体核型：46,XY[20]。应用二代测序（NGS）对267种血液系统疾病基因突变进行筛查：骨髓标本检测到与疾病密切相关的热点突变基因：KIT，突变位置：exon17，核苷酸改变：c.2447A>T，氨基酸改变：p.D816V，突变频率：37％；SF3B1，突变位置：exon14，核苷酸改变：c.1997A>G，氨基酸改变：p.K666T，突变频率：45％；以及ASXL1、NF1、H1-5和SETBP1基因突变。白血病43种融合基因筛查均阴性（包括BCR::ABL、ETV6::ABL1融合基因，PDGFRA、PDGFRB、FGFR1、JAK2、FLT3基因重排等）。腹部超声：脾脏长22.2 cm，厚6.6 cm，肋缘下7.9 cm×6.0 cm。促红细胞生成素（EPO）：300.59 mIU/ml。患者肝肾功能、生化、免疫等其他检查均无异常。诊断：SM合并MDS/MPN-非特指型，采用进展型系统性肥大细胞增多症（AdvSM）的突变校正危险度评分3分（高危）。

**图1:**
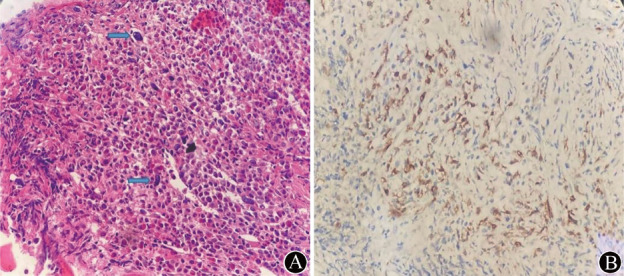
系统性肥大细胞增多症合并骨髓增生异常综合征/骨髓增殖性肿瘤患者骨髓病理结果（×40） **A** HE染色示增生极度活跃，箭头所指为胞体小的巨核细胞及裸核巨核细胞；**B** 免疫组织化学染色示CD117阳性的肥大细胞

患者诊断明确后，拒绝行异基因造血干细胞移植（allo-HSCT），输注红细胞3.5 U后，经患者知情同意后给予阿伐替尼（200 mg/d，口服）联合重组人EPO（10 000 IU/次，皮下注射，隔日1次）治疗。治疗1个月后患者血液学改善。血常规：HGB 101 g/L，WBC 2.87×10^9^/L，PLT 382×10^9^/L，脾脏较前缩小，腹部超声示脾脏长17.5 cm，厚6.1 cm，肋缘下4.8 cm×4.7 cm。治疗6个月后患者达到血液学完全缓解（CR）：HGB 125 g/L，WBC 6.06×10^9^/L，中性粒细胞绝对值（ANC）4.34×10^9^/L，PLT 398×10^9^/L。8个月后患者达到最佳临床疗效，血液学持续缓解，脾脏大小恢复大致正常，腹部超声示脾脏长14.4 cm，厚5.3 cm，肋缘下0 cm。8个月时应用NGS进行血液系统疾病基因突变筛查：未检测到KIT基因突变，检测到与疾病密切相关的热点突变基因为：SF3B1、ASXL1和NF1基因突变。患者治疗12个月外周血细胞计数变化见图2。患者应用阿伐替尼治疗期间出现眼睑水肿、直接胆红素和间接胆红素增高的非血液学不良事件，考虑与阿伐替尼药物相关，适时调整阿伐替尼剂量（100～200 mg/d）并予腺苷蛋氨酸等退黄药物对症治疗后略好转；EPO根据患者HGB调整剂量或停用。患者应用阿伐替尼治疗持续血液学缓解19个月后，再次出现贫血，白细胞增高；血常规：RBC 2.14×10^12^/L，HGB 78 g/L，WBC 48.32×10^9^/L，PLT 99×10^9^/L。外周血涂片分类计数：中性中幼粒细胞0.150，中性晚幼粒细胞0.060，中性杆状核粒细胞0.060，中性分叶核粒细胞0.380，嗜碱性粒细胞0.010，成熟淋巴细胞0.080，成熟单核细胞0.260。髂骨穿刺骨髓涂片分类：增生明显活跃，粒系0.475，红系0.265，成熟单核细胞0.140，原始细胞占0.010，粒系形态大致正常，红系可见巨幼样变和多核红细胞；全片共见巨核细胞161个。骨髓组织病理学检查：骨髓增生极度活跃（约90％），粒红比例大致正常，粒系各阶段细胞可见，红系以中晚幼红细胞为主，单核细胞散在分布，巨核细胞不少，分叶核为主，网状纤维染色MF-0级，免疫组化未见异常肥大细胞增多。铁染色：环形铁粒幼红细胞阳性率35％。染色体核型：46,XY[20]。应用NGS进行血液系统疾病基因突变筛查，未检测到KIT基因突变，检测到与疾病密切相关的突变基因为SF3B1、ASXL1、H1-5、NF1和SETBP1基因突变，治疗前后基因突变频率变化见图3。患者进展为慢性粒-单核细胞白血病1型（CMML-1型），患者拒绝行allo-HSCT，地西他滨去甲基化治疗3个月后因心功能衰竭死亡。

**图2:**
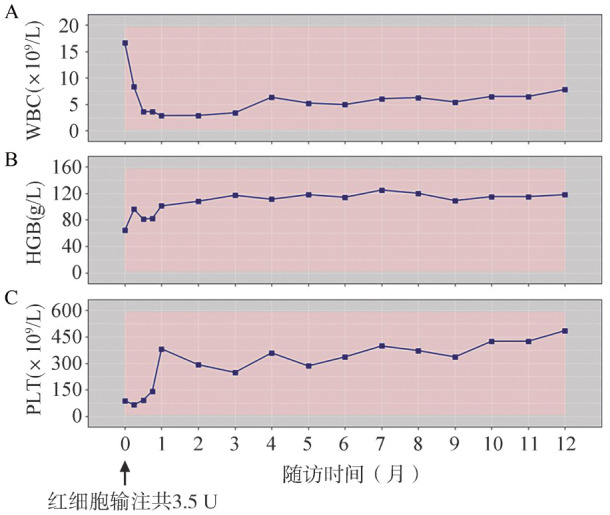
系统性肥大细胞增多症合并骨髓增生异常综合征/骨髓增殖性肿瘤患者接受阿伐替尼治疗后外周血细胞计数变化

**图3:**
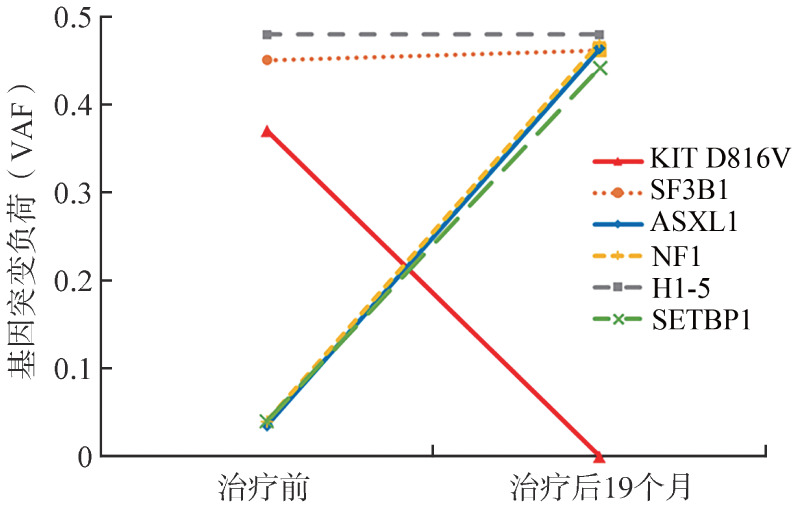
系统性肥大细胞增多症合并骨髓增生异常综合征/骨髓增殖性肿瘤患者阿伐替尼治疗前后基因突变频率变化

## 讨论及文献复习

肥大细胞增多症是异常肥大细胞在一个或多个组织器官中过度增殖、浸润导致的一种罕见的异质性肿瘤[2]。第五版WHO分型标准将肥大细胞增多症分为：皮肤型肥大细胞增多症（CM）、SM和肥大细胞肉瘤（MCS）三个亚型[2]–[3]。SM可进一步被划分为六种类型：惰性系统性肥大细胞增多症（ISM）、冒烟型系统性肥大细胞增多症（SSM）、骨髓肥大细胞增多症（BMM）、系统性肥大细胞增多症合并相关血液肿瘤（SM-AHN）、肥大细胞白血病（MCL）、侵袭性系统性肥大细胞增多症（ASM）[3]。在SM的所有类型中，ASM、SM-AHN和MCL三者被归为AdvSM[3]。在诊断SM-AHN时，需同时符合SM的诊断标准和相关血液肿瘤的诊断标准；有时由于AHN细胞大量浸润，极易掩盖肥大细胞的存在，进而干扰SM-AHN的诊断，需通过降细胞治疗后才能确立SM-AHN的诊断[2]。

现有研究已表明肥大细胞增多症与KIT基因突变相关。KIT（CD117）受体是KIT原癌基因的一种Ⅲ型受体酪氨酸激酶，当其发生突变时，受体磷酸转移酶结构域（phosphortransferase domain，PTD）的构象改变，可与干细胞因子（SCF）结合形成二聚体，激活下游MAPK、PI3K和JAK-STAT通路，使肥大细胞异常增殖从而引发局部反应或多系统症状[4]。KIT D816V是AdvSM发生的重要突变位点，且超过90％以上的SM患者携带该突变。羟基脲、干扰素-α和克拉屈滨等是临床上治疗AdvSM的传统药物，能改善SM相关症状，但此类药物靶向性差且对肥大细胞无选择性抑制作用[5]。甲磺酸伊马替尼、达沙替尼等传统酪氨酸激酶抑制剂对KIT D816V突变的SM无效；而对无KIT D816V突变的SM可能有一定疗效。Barozzi等[6]曾报道1例应用伊马替尼成功治疗无KIT D816V突变的SM合并MDS/MPN患者。阿伐替尼是一种新型选择性酪氨酸激酶抑制剂，能强效和高选择性抑制KIT D816V、PDGFRA D842V突变[7]。EXPLORER研究通过Ⅰ期临床试验评估阿伐替尼在成人AdvSM患者中的安全性和有效性：在69例AdvSM患者中，53例可评估治疗反应，总体有效率（overall response rate，ORR）为75％（40例），其中19例（36％）达到CR或CR伴部分血液学缓解（CRh），18例（34％）达到部分缓解（PR），3例（6％）达到临床改善（CI）[8]。2021年阿伐替尼治疗AdvSM的Ⅱ期临床试验（PATHFINDER）的中期分析结果[9]显示，88％（44/50）的患者骨髓肥大细胞较基线减少≥50％，60％（30/50）的患者骨髓肥大细胞聚集物完全清除；93％（54/58）的患者血清胰蛋白酶水平较基线下降≥50％；60％（33/55）的患者外周血KIT D816V的突变频率较基线降低≥50％，35％（19/55）的患者VAF<1％。32例可评估疗效［基线时有确定的SM诱导器官损害（C-发现）］的患者中，ORR为75％（24例），其中6例（19％）达到CRh，10例（31％）达到PR，8例（25％）达到CI。这32例患者中，ORR与基线时是否存在SRSF2/ASXL1/RUNX1（S/A/R）突变无关。基于上述临床试验的结果，美国食品药品监督管理局（FDA）和欧洲药品管理局（EMA）分别于2021年6月和2022年3月批准阿伐替尼用于治疗成人AdvSM（包括ASM、SM-AHN和MCL），推荐剂量为200 mg/d口服。

目前虽然没有阿伐替尼与最佳治疗方法（best available therapy，BAT）的随机对照试验，但一项多中心、回顾性队列研究比较了接受BAT（50％患者接受米哚妥林治疗，25％患者曾接受克拉屈滨治疗）的AdvSM患者与接受阿伐替尼治疗的Ⅰ、Ⅱ期临床试验患者的数据，证实了阿伐替尼治疗AdvSM的长期疗效[10]。与BAT组的患者相比，阿伐替尼组患者总生存（overall survival，OS）时间显著延长，阿伐替尼组与BAT组中位OS时间分别为49.0个月、26.8个月（*P*＝0.004）；与BAT组的患者相比，阿伐替尼组治疗持续（duration of therapy，DOT）时间更长，中位DOT时间分别为23.8个月、5.4个月（*P*<0.001）。目前亦没有阿伐替尼与米哚妥林治疗AdvSM的随机对照研究，一项系统综述比较了阿伐替尼与米哚妥林治疗AdvSM的疗效：阿伐替尼和米哚妥林治疗AdvSM调整后的ORR和CR的*OR*值分别为4.06（95％*CI*：3.09～5.33）和9.56（95％*CI*：0.97～93.81）。阿伐替尼与米哚妥林调整后的OS的*HR*值为0.44（95％*CI*：0.25～0.76）；与米哚妥林相比，阿伐替尼提高了AdvSM患者生存率和有效率[11]。

SM-AHN占所有AdvSM的70％～75％，大多数AHN来源于髓系肿瘤。CMML最常见，约占AHN的40％；其他包括MDS、MPN、慢性嗜酸性粒细胞白血病、MDS/MPN不能分类和急性髓系白血病（AML）[12]。SM-AHN可仅表现为骨髓形态上的轻度异常。分子和细胞遗传学异常通常有助于评估潜在的AHN。例如，SRSF2、ASXL1、TET2和（或）RAS通路的突变可能提示潜在的CMML或MDS/MPN，而JAK2、CALR或CSF3R的突变可能提示潜在的MPN[13]。大多数的成人AdvSM患者OS期短、预后差，欧洲肥大细胞增多症研究网络（ECNM）的登记数据显示ASM、SM-AHN、MCL的中位OS期分别为5.7、2.9和1.9年[14]。我国亦有研究表明，SM伴t（8;21）AML与KIT D816突变的t（8;21）AML具有相似的临床特征和分子学特征，是一类具有难治和易复发趋势不良预后的高危AML亚型[15]；而阿伐替尼与其他药物的联合治疗KIT D816突变的t（8;21）AML取得较好疗效[16]。针对SM-AHN患者的预后评估，可采用AdvSM的突变校正危险度评分（Mutation-Adjusted Risk Score，MARS）进行危险度分层：不良预后因素包括，年龄>60岁（积1分），HGB<100 g/L（积1分），PLT<100×10^9^/L（积1分），存在SRSF2/ASXL1/RUNX1（S/A/R）中的一种突变（积1分），≥2个S/A/R突变（积2分）；低危：0～1分；中危-1：2分；高危：3～5分[17]。

尽管强效KIT抑制剂（阿伐替尼等）能使SM达到比以往更持久的分子学缓解，但其对AHN疗效的影响仍不确定。在阿伐替尼Ⅰ期临床研究（EXPLORER研究）中，14例SM-AHN患者出现了疾病进展，其中10例患者与AHN进展相关。这些AHN进展与获得NRAS、GATA2、SRSF2、NPM1、SETBP1或CBL的突变相关，而与KIT D816V突变负荷的增加无关，表明出现了克隆演化[8]。AHN的治疗方法及其疗效因AHN的类型不同而有很大的差异。是先治疗SM还是先治疗AHN，取决于患者临床症状的主要原因和器官受累（C-发现）。由于使用选择性KIT抑制剂治疗SM比使用去甲基化药物治疗MDS、MDS/MPN或CMML更方便和有效，在诊断SM-AHN伊始便开始SM治疗并评估症状改善可能更合适。在这些患者中，应密切监测KIT D816V突变负荷并应用NGS监测克隆演化，以便密切地跟踪AHN变化。目前有观点认为，当SM经KIT抑制剂治疗后取得完全病理学缓解时（如检测不到KIT突变或骨髓无明显的异常肥大细胞），即应该开始AHN的治疗，而不是等到AHN进展时再治疗。如果AHN是一种高危的髓系肿瘤，如AML、伴有原始细胞增多的MDS或高危的MPN，那么通常需要优先治疗AHN，或者同时治疗SM和AHN。在SM-AHN患者中，将KIT抑制剂与其他抗肿瘤药物联合使用的数据非常有限。骨髓抑制程度的增加是我们主要关注的问题之一。在缺乏安全性数据的情况下，将AHN的治疗方法与KIT抑制剂联合时，谨慎的做法是以较低剂量开始联合治疗，以评估耐受性，然后密切监测不良反应并逐渐增加到最佳剂量[13]。

allo-HSCT是目前唯一可能治愈AdvSM的治疗方法。Ustun等[18]报道了一项allo-HSCT治疗AdvSM的回顾性研究，共纳入了57例患者（SM-AHN 38例，ASM 7例，MCL12例），所有AdvSM患者的ORR为70％，其中28％患者达到CR；所有38例SM-AHN患者均获得CR（100％），但随后有26％的患者AHN复发；所有患者的3年OS率为57％（SM-AHN为74％，ASM为43％，MCL为17％）。目前针对AdvSM的最佳移植时机尚无定论。既往的观点认为AdvSM患者应尽早行allo-HSCT，尤其是急性MCL患者、伴有高危髓系肿瘤或出现高危细胞遗传学和分子学克隆演化的AHN患者[19]。随着KIT抑制剂治疗SM能取得持续CR，目前越来越倾向于allo-HSCT的适应证和时机主要由合并的AHN情况决定。国内有研究报道，应用阿伐替尼治疗KIT突变的t（8;21）AML allo-HSCT后复发的患者，取得CR[20]。其后王娟等[21]报道了allo-HSCT后序贯阿伐替尼治疗SM伴RUNX1∷RUNX1T1阳性AML 2例，这两例患者移植后KIT D816V突变和RUNX1∷RUNX1T1/ABL均阴性，嵌合体均为完全嵌合。截至随访终点，2例患者无病生存时间分别为26.3、10.0个月。目前应用阿伐替尼联合allo-HSCT治疗SM-AHN的经验较少；上述研究报道提示，用阿伐替尼去除SM成分可能是一种有效的移植前策略；同样，allo-HSCT后序贯阿伐替尼亦可能作为SM-AHN有效的维持治疗方法。

本例患者在初诊时骨髓病理可见肥大细胞，呈多灶性致密浸润，梭形的肥大细胞比例明显增高；骨髓中肥大细胞表达CD20和CD30；骨髓中检测到KIT D816V突变（VAF 37％），符合SM诊断标准。该患者同时外周血表现为白细胞增高，贫血，单核细胞比例和绝对值未见明显增高；明显的脾肿大，骨髓形态示红系（环状铁粒幼红细胞增多）和巨核细胞形态异常，粒系未见形态异常；且检测到SF3B1、ASXL1、NF1和H1-5等MDS和MPN常见基因突变，符合MDS/MPN-非特指型，最终诊断为：SM-AHN。采用AdvSM的突变校正危险度评分为高危，患者拒绝行allo-HSCT，给予阿伐替尼200 mg/d口服以及EPO治疗。治疗1个月后患者增高的白细胞下降，贫血改善，脾脏明显缩小，展现出临床疗效，继续给予上述药物治疗；治疗8个月时患者血常规完全正常，脾脏大小恢复至正常，达到IWG-MRT-ECNM共识的CR，且未检测到KIT基因突变。持续治疗19个月后患者血液肿瘤进展为CMML，并出现克隆演化（ASXL1、NF1和SETBP1突变频率明显增高）；但骨髓未见肥大细胞增多，未检测到KIT基因突变，提示患者SM疾病得到持续CR，最终因AHN疾病进展而死亡。

综上，本文通过1例阿伐替尼治疗SM合并MDS/MPN，SM持续CR，最终因AHN疾病进展而死亡的病例，指导临床对SM-AHN的诊治。虽然新的靶向药物阿伐替尼显著提高了SM的疗效，但对于SM-AHN患者，其伴随的血液肿瘤的疗效可能是影响其OS期和无进展生存期的主要因素，其相关影响有待进一步研究证实。在AHN为MDS/MPN时，阿伐替尼联合去甲基化药物治疗具有一定的可行性，其疗效有待进一步评估；考虑到去甲基化药物可导致严重血小板减少，而在严重血小板减少情况下阿伐替尼增加了颅内出血的风险，因此阿伐替尼与去甲基化药物同时联合应用的可能性有待商榷；而根据患者临床表现，阿伐替尼与去甲基化药物交替治疗可能是更好的治疗策略[12]。在AdvSM中，allo-HSCT仍然是一个合理的选择，特别是在AHN中具有高危特征的患者中。对于这类患者用阿伐替尼去除SM成分可能是一种有效的移植前策略；allo-HSCT后序贯阿伐替尼亦可能作为SM-AHN有效的维持治疗方法。目前应用阿伐替尼联合allo-HSCT治疗SM-AHN多为个案报道，有待于今后临床研究进行证实，以期提高AdvSM患者的整体疗效。

## References

[1] Pardanani A (2023). Systemic mastocytosis in adults: 2023 update on diagnosis, risk stratification and management[J]. Am J Hematol.

[2] 中华医学会血液学分会实验诊断学组, 中国肥大细胞增多症协作网络 (2022). 成人系统性肥大细胞增多症诊断与治疗中国指南(2022年版)[J]. 中华血液学杂志.

[3] Khoury JD, Solary E, Abla O (2022). The 5th edition of the World Health Organization Classification of Haematolymphoid Tumours: Myeloid and Histiocytic/Dendritic Neoplasms[J]. Leukemia.

[4] Chatterjee A, Ghosh J, Kapur R (2015). Mastocytosis: a mutated KIT receptor induced myeloproliferative disorder[J]. Oncotarget.

[5] Lim KH, Pardanani A, Butterfield JH (2009). Cytoreductive therapy in 108 adults with systemic mastocytosis: Outcome analysis and response prediction during treatment with interferon-alpha, hydroxyurea, imatinib mesylate or 2-chlorodeoxyadenosine[J]. Am J Hematol.

[6] Barozzi E, Bucelli C, Grifoni FI (2021). Successful Imatinib Treatment for Systemic Mastocytosis Associated With Myelodysplastic/Myeloproliferative Neoplasm: Report of a Case and Literature Review[J]. Front Oncol.

[7] Dhillon S (2020). Avapritinib: First Approval[J]. Drugs.

[8] DeAngelo DJ, Radia DH, George TI (2021). Safety and efficacy of avapritinib in advanced systemic mastocytosis: the phase 1 EXPLORER trial[J]. Nat Med.

[9] Gotlib J, Reiter A, Radia DH (2021). Efficacy and safety of avapritinib in advanced systemic mastocytosis: interim analysis of the phase 2 PATHFINDER trial[J]. Nat Med.

[10] Reiter A, Gotlib J, Álvarez-Twose I (2022). Efficacy of avapritinib versus best available therapy in the treatment of advanced systemic mastocytosis[J]. Leukemia.

[11] Pilkington H, Smith S, Roskell N (2022). Indirect treatment comparisons of avapritinib versus midostaurin for patients with advanced systemic mastocytosis[J]. Future Oncol.

[12] Gotlib J, Reiter A, DeAngelo DJ (2022). Avapritinib for advanced systemic mastocytosis[J]. Blood.

[13] Tashi T, Deininger MW (2023). Management of Advanced Systemic Mastocytosis and Associated Myeloid Neoplasms[J]. Immunol Allergy Clin North Am.

[14] Sperr WR, Kundi M, Alvarez-Twose I (2019). International prognostic scoring system for mastocytosis (IPSM): a retrospective cohort study[J]. Lancet Haematol.

[15] Zhang Z, Yin J, Lian G (2024). A multicenter retrospective comparison between systemic mastocytosis with t(8;21) AML and KIT mutant t(8;21) AML[J]. Blood Adv.

[16] Yin J, Zhu F, Zhang ZB (2022). Rapid and deep response to avapritinib in heavily treated acute myeloid leukemia with t (8;21) and KIT mutation[J]. Ann Hematol.

[17] Jawhar M, Schwaab J, Álvarez-Twose I (2019). MARS: Mutation-Adjusted Risk Score for Advanced Systemic Mastocytosis[J]. J Clin Oncol.

[18] Ustun C, Reiter A, Scott BL (2014). Hematopoietic stem-cell transplantation for advanced systemic mastocytosis[J]. J Clin Oncol.

[19] Ustun C, Gotlib J, Popat U (2016). Consensus Opinion on Allogeneic Hematopoietic Cell Transplantation in Advanced Systemic Mastocytosis[J]. Biol Blood Marrow Transplant.

[20] Xue S, Huang W, Liu F (2022). Rapid response to avapritinib of acute myeloid leukemia with t(8;21) and KIT mutation relapse post allo-HSCT[J]. Leuk Lymphoma.

[21] 王 娟, 祖 璎玲, 桂 瑞瑞 (2024). 异基因造血干细胞移植后序贯阿伐替尼治疗系统性肥大细胞增多症伴RUNX1-RUNX1T1阳性急性髓系白血病2例并文献复习[J]. 中华血液学杂志.

